# Evaluating the efficacy of an integrated smoking cessation intervention for mental health patients: study protocol for a randomised controlled trial

**DOI:** 10.1186/1745-6215-15-266

**Published:** 2014-07-05

**Authors:** Alexandra P Metse, Jenny A Bowman, Paula Wye, Emily Stockings, Maree Adams, Richard Clancy, Margarett Terry, Luke Wolfenden, Megan Freund, John Allan, Judith J Prochaska, John Wiggers

**Affiliations:** 1 University of Newcastle, University Drive, Callaghan, NSW 2308, Australia; 2 Hunter Medical Research Institute, Lot 1 Kookaburra Circuit, New Lambton Heights, NSW 2305, Australia; 3 Hunter New England Population Health, Longworth Ave, Wallsend, NSW 2287, Australia; 4 Centre for Translational Neuroscience and Mental Health, Mater Hospital, Cnr Edith and Platt Streets, Waratah, NSW 2298, Australia; 5 Mental Health and Substance Use Service, Mater Hospital, Cnr Edith and Platt Streets, Waratah, NSW 2298, Australia; 6 Mental Health and Drug and Alcohol Office, NSW Department of Health, 73 Miller Street, North Sydney, NSW 2060, Australia; 7 Stanford Prevention Research Centre, Medical School Office Building, 1265 Welch Road, Mail Code 5411, Stanford, CA 94305-5411, USA

**Keywords:** Smoking cessation, Mental illness, Inpatient, Community, Multimodal intervention

## Abstract

**Background:**

Smoking rates, and associated negative health outcomes, are disproportionately high among people with mental illness compared to the general population. Smoke-free policies within mental health hospitals can positively impact on patients’ motivation and self-efficacy to address their smoking. However, without post-discharge support, preadmission smoking behaviours typically resume. This protocol describes a randomised controlled trial that aims to assess the efficacy of linking mental health inpatients to community-based smoking cessation supports upon discharge as a means of reducing smoking prevalence.

**Methods/Design:**

Eight hundred participants with acute mental illness will be recruited into the randomised controlled trial whilst inpatients at one of four psychiatric inpatient facilities in the state of New South Wales, Australia. After completing a baseline interview, participants will be randomly allocated to receive either: ‘Supported Care’, a multimodal smoking cessation intervention; or ‘Normal Care’, consisting of existing hospital care only. The ‘Supported Care’ intervention will consist of a brief motivational interview and a package of self-help material for abstaining from smoking whilst in hospital, and, following discharge, 16 weeks of motivational telephone-based counselling, 12 weeks of free nicotine replacement therapy, and a referral to the Quitline. Data will be collected at 1, 6 and 12 months post-discharge via computer-assisted telephone interview. The primary outcomes are abstinence from smoking (7-day point prevalence and prolonged cessation), and secondary outcomes comprise daily cigarette consumption, nicotine dependence, quit attempts, and readiness to change smoking behaviour.

**Discussion:**

If shown to be effective, the study will provide evidence in support of systemic changes in the provision of smoking cessation care to patients following discharge from psychiatric inpatient facilities.

**Trial registration:**

Australian New Zealand Clinical Trials Registry ANZTCN: ACTRN12612001042831. Date registered: 28 September 2012.

## Background

Tobacco use is the second leading cause of modifiable morbidity and mortality worldwide [1]. In Australia, the prevalence of smoking in the general population has halved over the past three decades to approximately 15% [2]. However, depending on diagnosis and setting, between 33% and 90% of people with mental illness continue to smoke [3,4]. Patients with psychotic disorders have among the highest smoking prevalence (74% to 88%) [5]. Smokers with a mental illness also smoke more heavily, are more nicotine dependent and find it more difficult to quit compared to the general population [5-7]. As a consequence, smokers with a mental illness are more likely to have a chronic disease and to have a shorter life expectancy [8-11].

For smokers in the general population [12,13] and for those with a mental illness [14,15] multimodal smoking cessation interventions, utilising both pharmacological interventions (such as nicotine replacement therapy (NRT)) and psychosocial supports (such as behavioural counselling and self-help materials), have demonstrated efficacy in increasing the likelihood of quitting successfully. Interventions of a longer duration and greater intensity are more efficacious for both groups of smokers [11,16-18]. Further, neither making a quit attempt nor successfully ceasing smoking has been found to negatively impact on [14], and may even improve [19-22], psychiatric health.

Hospitals have been recognised as a key setting for initiating the delivery of smoking cessation care [17,20,23,24]. The implementation of total smoking bans in mental health hospitals [11,25] and the ensuing need to manage inpatient nicotine withdrawal [12,26] reinforces the need to provide smoking cessation treatment in this setting [27]. Evidence suggests that the initiation of smoking cessation treatment in the inpatient psychiatric setting also increases patient motivation and self-efficacy to change their smoking behaviour, both as an inpatient and following discharge [28]. However, as is the case in general medical settings [29], evidence from psychiatric settings suggests that without post-discharge support smoking is likely to return to preadmission levels within 2 weeks [30]. Such findings suggest a need for adequate and consistent support to encourage a sustained quit attempt and prevent relapse [30-32]. Systematic review evidence from general medical settings suggests that multimodal post-discharge cessation support of at least 4 weeks in length or greater can result in higher rates of successful smoking cessation [17].

A recent randomised controlled trial has demonstrated the effectiveness of linking mental health inpatients to community-based smoking support following discharge. Prochaska and colleagues [20] reported a significant difference in point prevalence abstinence at 18 months post-discharge; 20% and 7.7% for intervention and control conditions, respectively. Over a period of up to 6 months post-discharge, intervention group participants were able to take up both pharmacological (NRT for a total duration of up to 10 weeks) and psychosocial (face-to-face, computer-based, and self-directed reading) smoking cessation support, tailored to their readiness to stop smoking. An opportunity may exist to enhance and extend such positive findings by providing multi-modal smoking cessation support to all smokers, proactively and intensively, immediately following discharge, regardless of assessed readiness to change.

To assess the feasibility of such an approach we conducted a pilot randomised controlled trial of an intervention consisting of the provision of psychological and pharmacological support to smokers admitted to one inpatient psychiatric facility. Inpatient smokers were randomly allocated to a multimodal smoking cessation intervention or treatment-as-usual control. Smoking cessation treatment was initiated for all smokers during admission and continued for a 4-month period post-discharge with assessments made at 1 week and at 2, 4 and 6 months [33]. At the end of treatment (4 months), participants in the intervention group had significantly higher rates of 7-day point prevalence abstinence than controls (11.5% versus 2%), but this difference was not sustained at 6 months follow-up [34]. Based on the findings of these previous studies, the objective of this study is to conduct a randomised controlled trial to test the effectiveness of a multi-modal smoking cessation intervention, initiated within mental health inpatient facilities for all smokers and continued post-discharge, on 12-month post-discharge smoking cessation rates.

## Methods/Design

### Study design and setting

**Figure 1:**
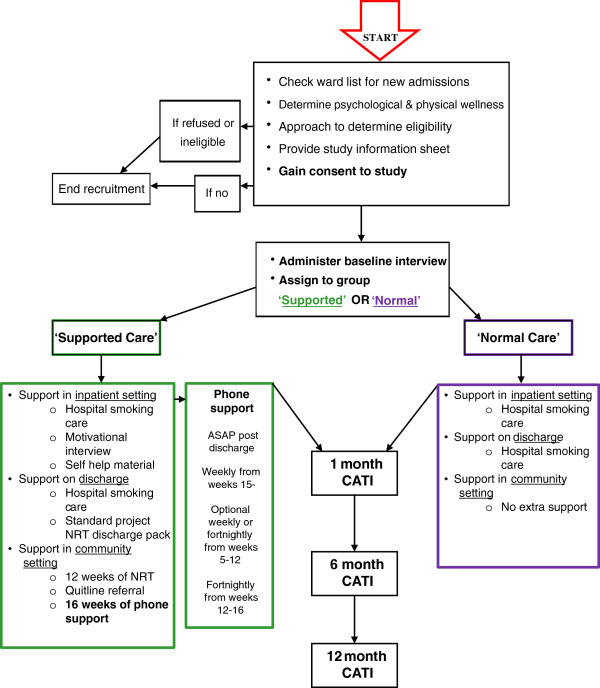
**Study design.** ASAP, as soon as possible; CATI, computer-assisted telephone interview; NRT, nicotine replacement therapy.

A randomised controlled trial with blinded follow-up will be conducted (Figure 1). Participants will be recruited as inpatients at one of four acute mental health facilities within one Local Health District in New South Wales, Australia. At the time of recruitment, participants will be randomly allocated to either an intervention (Supported Care) or control (Normal Care) group. Participants allocated to the Supported Care condition will receive a brief motivational interview and a package of self-help material for abstaining from smoking whilst in hospital, and intensive psychosocial and pharmacological support for 16 weeks upon hospital discharge. Participants in the Normal Care condition will receive standard hospital and discharge smoking cessation care.

Follow-up assessments will be conducted at 1, 6 and 12 months post-discharge for both conditions. The primary outcome measures comprise 7-day point prevalence of smoking abstinence and prolonged smoking abstinence. Secondary outcome measures will include number of cigarettes smoked per day, nicotine dependence, quit attempts, and readiness to change smoking behaviour. The effect of the intervention will be assessed by comparing primary and secondary outcome measures between intervention and control groups at each follow-up point.

Ethics approval for the study has been obtained from the Hunter New England Human Research Ethics Committee, HNEHREC reference no.: 11/12/14/4.02, and the University of Newcastle Human Research Ethics Committee, reference no.: H-2012-0061. The trial is registered on the Australian New Zealand Clinical Trials Registry ACTRN12612001042831. Date registered: 28 September 2012.

### Recruitment process and inclusion criteria

Research staff (independent of the hospitals), all of whom will receive standardised training in mental illness and its impacts, will approach nurse unit managers in the four units on a daily basis to identify newly admitted patients. The nurse unit managers will advise whether individual patients are sufficiently psychiatrically stabilised and capable of completing the baseline interview. If so, patients will be approached to determine if they meet the eligibility criteria to participate in the study. Patients will be eligible if they: are a current smoker (smoked cigarettes daily, weekly or less than weekly in the month prior to admission); 18 years of age or above; understand the research, have capacity to ask and answer questions in relation to the benefits and potential harms (that is, have capacity to give informed consent); and are willing to provide contact details (including phone number and mailing address) to facilitate communication once discharged. No exclusion criteria will be applied. Patients who meet the eligibility criteria will be given a detailed explanation of the project requirements and invited to participate in the trial. Informed consent will be gained from all participants. Patients who elect not to take part, and who are subsequently readmitted to the facility during the recruitment phase, will be re-approached and re-offered the opportunity to participate. Patients will only be recruited once, regardless of the number of readmissions within the study recruitment phase. Eight hundred participants will be recruited over a period of 18 months.

### Randomisation, allocation concealment and sequence generation

A random allocation sequence using permuted block randomisation (with a block size of ten) will be generated by a statistician independent of the project prior to the commencement of recruitment. Randomisation will be carried out separately by site and stratified by diagnosis type to account for differences in smoking levels and nicotine dependence (psychosis/non-psychosis type diagnosis) [5,35]. Participant allocation to group (1:1 ratio) will occur immediately post-baseline data collection. Research staff will provide participants with a sequentially numbered, opaque, sealed envelope. The envelope will contain a coloured piece of paper that indicates allocation to either the Supported Care or Normal Care condition. Research staff involved in recruitment and outcome data collection will be blind to the order of patient condition assignment.

### Data collection procedures

For eligible and consenting patients, baseline data will be collected during admission via a face-to-face baseline interview, administered by research staff in a quiet area of the unit. The baseline data collection interview will be developed based on previous pilot research undertaken by the research team [33]. After the completion of the baseline interview, clinical and demographic information will be collected by research staff from patient medical records.

Follow-up data will be collected via computer-assisted telephone interviews (CATI) from all participants at 1, 6 and 12 months post-discharge. In addition, process data will be collected during support calls to assess intervention uptake.

### Intervention

#### Normal care

Participants allocated to the Normal Care condition will receive standard hospital smoking care only, as routinely delivered by hospital staff. This may include brief advice to quit, provision of NRT whilst admitted to the inpatient facility, up to 3 days supply of NRT upon discharge, and/or a referral to Quitline as per mandatory local area health guidelines [26,36]. Previous research suggests that standard hospital care is likely to be limited [37,38].

#### Supported care

In addition to standard smoking care provided by the hospital, participants allocated to the Supported Care condition will be provided with a range of evidence-based smoking cessation supports [12,18]. Following the baseline interview, supported care participants will be provided with smoking cessation written self-help material [39], including information tailored to people with mental illness [40-42] and NRT usage advice, and a brief motivational interview (10 to 15 minutes) designed to evoke ambivalence regarding smoking behaviour and reinforce positive intentions to address smoking [43]. Content of the motivational interview will encourage consideration of: lifestyle behaviours; pros and cons of both continuing to smoke and quitting; impact of smoking on life in 5 years from the time of interview; and importance of and confidence to quit [43].

Upon discharge from hospital, all Supported Care participants will receive a standard project pack of NRT, which contains: seven nicotine patches (21 mg); one nicotine inhaler with six cartridges (10 mg); 30 pieces of nicotine gum (4 mg); and 20 nicotine lozenges (4 mg). NRT discharge packs will also contain a brief tip sheet for NRT use and the management of NRT side effects, as well as a project reminder card outlining each aspect of the intervention. Within participating units, the process for providing discharge NRT packs will be negotiated in consultation with nurse unit managers and treating staff, in accordance with usual protocols for discharge, and storage of medications. The participant’s general practitioner and, if applicable, community mental health team will be notified of their clients’ involvement in the project, via the standard and automated delivery of discharge records to relevant health professionals, and asked to support their participation in the project [17].

Once discharged from hospital, participants in the Supported Care arm will receive up to 16 weeks of psychosocial [44] and 12 weeks of pharmacological support [12,13,45]. Psychosocial support will initially be delivered through five weekly telephone support calls, followed by 7 weeks of weekly or fortnightly telephone support calls (tailored based on participants preference), tapering to 4 weeks of fortnightly support calls between weeks 12 and 16 (Figure 1). The content of the calls will be delivered utilising a framework of motivational interviewing techniques [46], and incorporate behavioural strategies to assist with addressing smoking. Support call staff will prompt participants to utilise the Quitline, and offer to send a Quitline referral on their behalf. Support calls will be delivered by research staff that either have a relevant allied health qualification and/or experience working with people with mental illness. All support callers will receive both standardised mental health training and ongoing motivational interviewing based training and supervision from an experienced clinical consultant.

Ongoing NRT provision will be offered by research staff to all supported care participants, with delivery by mail. The amount and frequency of NRT provided will vary by participant in accordance with personal preference, degree of nicotine dependence (measured using time to first cigarette) [47], and nicotine withdrawal symptoms [48]. Within the initial 12 weeks of support post-discharge, participants are able to optionally elect any combination of the following NRT products: patches (21, 15, 14 or 7 mg); lozenges and/or mini lozenges (2 and 4 mg); gum (a variety of flavours; 2 and 4 mg); inhalers (10 and 15 mg refill cartridges); oral spray (one dispenser contains 150 × 1 mg sprays); and oral strips (2.5 mg). Pharmacological support will be provided in accordance with an evidence-based, combination NRT algorithm aimed at smokers with a high level of nicotine dependence and who have more difficulty quitting [49].

### Measures

The primary and secondary outcome measures will be collected via CATI at 1, 6 and 12 months post-discharge.

#### Primary outcome measures

The primary outcome measure will be abstinence from smoking, both 7-day point prevalence and prolonged abstinence. Participants who report being abstinent from smoking for 7 days or greater will be required to submit a breath sample to validate their quit attempt. CATI interviewers will organise a time to carry out this procedure and project staff, blind to allocation group, will meet participants in a public place or their home as soon as can be arranged to verify abstinence. Breath samples will be taken using a Micro^+^ Smokerlyser (Bedfont Scientific Ltd, http://www.bedfont.com/smokerlyzer), where the exhaled carbon monoxide level must not exceed 6.99 ppm for the self- reported abstinence to be considered valid [50,51].

#### Secondary outcome measures

Secondary outcome measures will include number of cigarettes smoked per day, quit attempts made (number, duration, and supports used), nicotine dependence (assessed using the Fagerstrom Test for Nicotine Dependence) [47], and motivation and readiness to quit (Readiness and Motivation to Quit Smoking Questionnaire) [52].

### Process measures

#### All participants

Details regarding the delivery of any smoking-related care and/or support, received during the initial and any subsequent hospital admissions, will be collected by CATI staff at each of the three follow-up points.

#### Supported care participants only

As part of the motivational interview administered following assignment to supported care, participants will be asked to rank on a scale from 1 to 10 both importance of, and confidence to, quit smoking. To assess the degree of Supported Care intervention delivery, descriptive data will be collected during support calls by the designated support caller, including: the receipt and usage of discharge NRT pack; contact with, and perceived effectiveness of, Quitline support (if applicable); intensity and duration of support calls; dose, type, amount, usage and perceived effectiveness of NRT; side effects of NRT use; and nicotine withdrawal symptoms. Data assessing the degree of participant satisfaction with the intervention will also be collected by CATI interviewers after the completion of the 12-month follow-up.

#### Patient characteristics

Demographic and clinical information including age, gender, marital status, mental health diagnosis (primary and secondary), and Aboriginal and/or Torres Strait Islander status will be collected by recruitment staff from patient medical records, after the completion of the baseline interview. Education levels, employment status, identity as a smoker (items based on the PRIME theory of addiction [53]), and levels of perceived support to quit smoking from partner, family, friends, and relevant health professionals will be collected as part of the baseline interview. Other patient characteristics including alcohol consumption (Alcohol Use Disorders Identification Test) [54], psychological distress (the six-item version of the Kessler Psychological Distress Scale) [55], and perceived levels of stress (the four-item version of the Perceived Stress Scale) [56] will also be assessed at baseline and each of the follow-up points.

#### Intervention cost

Descriptive data on direct costs of the intervention will be collected as part of routine project delivery and administration throughout the project, and include all costs associated with inpatient and post-discharge intervention.

### Sample size

Considering 80% power with a 5% significance level, 332 participants per group will be required to detect a 4% difference (1% versus 5% for Normal and Supported Care participants, respectively) in prolonged abstinence at 12 months post-discharge. This sample size will also be adequate to detect a 10% difference in point prevalence abstinence at 12 months post-discharge [20].

### Statistical analysis

Data will be analysed using IBM SPSS Statistics 22 (International Business Machines Corporation, http://www-01.ibm.com/software/au/analytics/spss/).

Prior to any statistical analysis, baseline predictors of attrition will be examined. If it appears missing data are related to a measured aspect of the participants, those measures will be included as covariates in the hypothesis-testing models. The modelling strategy will allow the use of all collected data in our estimation. Sensitivity analyses will check that methods of dealing with missing data do not have a major impact on study conclusions. Outcome analyses, based on coding missing subjects as "smoking", will allow direct comparison of findings with the research literature.

#### Analysis of primary outcome measures

A linear model with estimation via Generalized Estimating Equations, using the logit link function given the dichotomous outcome variable, will be used to examine abstinence versus smoking status at 12 months by condition. The data collected at 1 and 6 months will also be examined; however, the 12-month follow-up will be the primary focus. Generalized Estimating Equations, a multivariate extension of generalised linear models, accounts for dependence of responses within individuals due to repeated measures. Any covariates identified in preliminary data analyses may be added to the model. Subgroup analyses will be considered, likely by dependence and diagnosis (psychosis versus non-psychosis) [5,57]. All analyses will be conducted using intent-to-treat principles, with participants retained in their originally assigned groups [58].

#### Analysis of secondary outcome measures

Chi square and *t*-test (or Mann–Whitney) analysis will be used to compare the number of quit attempts, nicotine dependence, cigarettes smoked per day, and motivation to change smoking behaviour between the intervention and control conditions. Descriptive statistics will be used to report process data.

## Discussion

Further research is needed to assess the effectiveness of a proactive and integrated approach to the provision of smoking cessation treatment to psychiatric inpatients immediately upon discharge, regardless of a patient’s assessed readiness to quit, in increasing abstinence and secondary smoking-related outcomes. This randomised controlled trial builds upon pilot work [33], and incorporates enhanced elements including a larger sample size and greater statistical power, longer assessment of outcomes to 12 months post-discharge, use of a more structured motivational interviewing framework in the delivery of psychosocial support, and a more comprehensive and flexible NRT protocol in the delivery of pharmacological support.

The conduct of this trial and its findings will substantively strengthen the base of evidence available to inform the development and delivery of smoking cessation treatment to persons with a mental illness. It will hence contribute to redressing the significant health and social inequities experienced by this population sub-group as a consequence of tobacco smoking.

## Trial status

Recruitment for this project commenced in October 2012, with a plan to proceed until April 2014. Follow-up data collection has commenced; it is anticipated that it will conclude in June 2015.

## Abbreviations

CATI: computer-assisted telephone interview; NRT: nicotine replacement therapy.

## Competing interests

The authors declare that they have no competing interests.

## Authors’ contributions

APM drafted the manuscript and participated in the conception, design and coordination of the study. JAB, PW, RC, ES, MA and JW helped draft the manuscript and participated in the conception, design and coordination of the study. MT, JJP, LW, MF and JA helped draft the manuscript and participated in the conception and design of the study. All authors critically revised and approved the final manuscript for publication.
